# Supplementary material for: Empirical methods for controlling false positives and estimating confidence in ChIP-Seq peaks
Source: BMC Bioinformatics. 2008 Dec 5;9:523. doi: 10.1186/1471-2105-9-523 (PMC2628906; doi:10.1186/1471-2105-9-523)
Supplement: Additional File 1 — A variety of html documents from the USeq web site detailing the available applications, their best usage, output file type descriptions, command line menus, etc. [file 1471-2105-9-523-S1.zip › USeqUserGuides/JavaDoc.zip!/JavaDoc/trans/main/ChromSet.html]

ChromSet


|  |  |  |
| --- | --- | --- |
| **Overview** \| **Package** \| **Class** \| **Use** \| **Tree** \| **Deprecated** \| **Index** \| **Help** | |  |
| **PREV CLASS**   **NEXT CLASS** | **FRAMES**    **NO FRAMES**     **All Classes** |  |
| SUMMARY: NESTED \| FIELD \| CONSTR \| METHOD | DETAIL: FIELD \| CONSTR \| METHOD |  |


---


## trans.main Class ChromSet

```
java.lang.Object
  trans.main.ChromSet
```

---

``` public class ChromSet extends java.lang.Object ```

---

**Constructor Summary**

`ChromSet()`


| **Method Summary** | |
| --- | --- |
| `java.io.File[]` | `getChipSetDirectories()` |
| `long` | `getNumberOligos()` |
| `java.io.File` | `getSaveDirectory()` |
| `boolean` | `isTreatmentReplica()` |
| `void` | `loadNumberOligos()` |
| `void` | `setChipSetDirectories(java.io.File[] chipSetDirectories)` |
| `void` | `setNumberOligos(long numberOligos)` |
| `void` | `setSaveDirectory(java.io.File saveDirectory)` |
| `void` | `setTreatmentReplica(boolean treatmentReplica)` |

**Methods inherited from class java.lang.Object**

`equals, getClass, hashCode, notify, notifyAll, toString, wait, wait, wait`

**Constructor Detail**

### ChromSet

```
public ChromSet()
```


**Method Detail**

### loadNumberOligos

```
public void loadNumberOligos()
```

---


### getChipSetDirectories

```
public java.io.File[] getChipSetDirectories()
```

---


### setChipSetDirectories

```
public void setChipSetDirectories(java.io.File[] chipSetDirectories)
```

---


### getNumberOligos

```
public long getNumberOligos()
```

---


### setNumberOligos

```
public void setNumberOligos(long numberOligos)
```

---


### getSaveDirectory

```
public java.io.File getSaveDirectory()
```

---


### setSaveDirectory

```
public void setSaveDirectory(java.io.File saveDirectory)
```

---


### isTreatmentReplica

```
public boolean isTreatmentReplica()
```

---


### setTreatmentReplica

```
public void setTreatmentReplica(boolean treatmentReplica)
```


---


|  |  |  |
| --- | --- | --- |
| **Overview** \| **Package** \| **Class** \| **Use** \| **Tree** \| **Deprecated** \| **Index** \| **Help** | |  |
| **PREV CLASS**   **NEXT CLASS** | **FRAMES**    **NO FRAMES**     **All Classes** |  |
| SUMMARY: NESTED \| FIELD \| CONSTR \| METHOD | DETAIL: FIELD \| CONSTR \| METHOD |  |


---
